# Decoding of Envelope vs. Fundamental Frequency During Complex Auditory Stream Segregation

**DOI:** 10.1162/nol_a_00013

**Published:** 2020-07-01

**Authors:** Keelin M. Greenlaw, Sebastian Puschmann, Emily B. J. Coffey

**Affiliations:** Department of Psychology, Concordia University, Montreal, QC, Canada; International Laboratory for Brain, Music and Sound Research (BRAMS); The Centre for Research on Brain, Language and Music (CRBLM); Institute of Psychology, University of Lübeck, Lübeck, Germany; Department of Psychology, Concordia University, Montreal, QC, Canada; International Laboratory for Brain, Music and Sound Research (BRAMS); The Centre for Research on Brain, Language and Music (CRBLM)

**Keywords:** auditory stream segregation, hearing-in-noise, pitch representation, reconstruction, speech-in-noise, neural decoding

## Abstract

Hearing-in-noise perception is a challenging task that is critical to human function, but how the brain accomplishes it is not well understood. A candidate mechanism proposes that the neural representation of an attended auditory stream is enhanced relative to background sound via a combination of bottom-up and top-down mechanisms. To date, few studies have compared neural representation and its task-related enhancement across frequency bands that carry different auditory information, such as a sound’s amplitude envelope (i.e., syllabic rate or rhythm; 1–9 Hz), and the fundamental frequency of periodic stimuli (i.e., pitch; >40 Hz). Furthermore, hearing-in-noise in the real world is frequently both messier and richer than the majority of tasks used in its study. In the present study, we use continuous sound excerpts that simultaneously offer predictive, visual, and spatial cues to help listeners separate the target from four acoustically similar simultaneously presented sound streams. We show that while both lower and higher frequency information about the entire sound stream is represented in the brain’s response, the to-be-attended sound stream is strongly enhanced only in the slower, lower frequency sound representations. These results are consistent with the hypothesis that attended sound representations are strengthened progressively at higher level, later processing stages, and that the interaction of multiple brain systems can aid in this process. Our findings contribute to our understanding of auditory stream separation in difficult, naturalistic listening conditions and demonstrate that pitch and envelope information can be decoded from single-channel EEG data.

## INTRODUCTION

Hearing-in-noise (HIN) is a complex and computationally challenging task that is critical to human function in social, educational, and vocational contexts. Anecdotally, our HIN skills can sometimes be strikingly effective, as when we catch a phrase from a familiar song on the radio and can suddenly perceive its entirety over the din of a crowded supermarket. More frequently, attending to a target sound stream in the presence of noise is perceived to be effortful and fatiguing (Heinrich, Schneider, & Craik, 2008; McGarrigle et al., 2014). Using compensatory mechanisms negatively impacts on other cognitive functions (Peelle, 2018; Wu, Stangl, Zhang, Perkins, & Eilers, 2016), with consequences for well-being (Eckert, Teubner-Rhodes, & Vaden, 2016). The large number of people affected, notably older adults (Anderson, Parbery-Clark, White-Schwoch, & Kraus, 2012; Anderson, Parbery-Clark, Yi, & Kraus, 2011) and some paediatric populations (Ziegler, Pech-Georgel, George, Alario, & Lorenzi, 2005), motivates efforts to clarify the neural mechanisms underlying this complex behaviour. Furthermore, noninvasive, low-cost, and pleasurable interventions such as musical training might improve HIN skills (Dubinsky, Wood, Nespoli, & Russo, 2019), but to realise their potential we require a better understanding of the degree to which language and musical processing that are critical for HIN perception rely on the same mechanisms (Särkämö, Altenmüller, Rodríguez-Fornells, & Peretz, 2016).

The brain appears to rely on both the quality of feed-forward, bottom-up encoding (Coffey, Chepesiuk, Herholz, Baillet, & Zatorre, 2017; Song, Skoe, Banai, & Kraus, 2011) and active top-down mechanisms to enhance or “sharpen” task-relevant acoustic features in the presence of competing sound (Bidet-Caulet et al., 2007; Du & Zatorre, 2017; Forte, Etard, & Reichenbach, 2017; Puschmann et al., 2017). Feed-forward encoding can be observed in the fidelity with which aspects of sound are encoded in the brain, whereas the contributions of top-down (or multimodal) factors can be teased out via experimental design (e.g., selective attention paradigms). Two frequency bands carry important acoustic information, the neural representations of which can be measured in time-resolved neuroimaging methods, such as EEG and MEG.

Lower frequency (i.e., ∼1–9 Hz) fluctuations convey information concerning the temporal envelope of sound, which is related to the rate of words, syllables, and phonemes in speech (Keitel, Gross, & Kayser, 2018) and also rhythmic elements in music (Harding, Sammler, Henry, Large, & Kotz, 2019). For continuous auditory input, such as natural speech or music, the neural response to the sound envelope can be assessed using linear mapping approaches (Crosse, Di Liberto, Bednar, & Lalor, 2016). Stimulus reconstruction (i.e., backward mapping from EEG/MEG data to the sound envelope) allows us to quantify the accuracy/robustness of the cortical envelope response (Ding & Simon, 2011). In the context of speech-in-noise, differences in the speech envelope response have been associated with individual differences in speech intelligibility (Ding & Simon, 2013). In selective listening paradigms, in which participants must attend to one of two or more sound streams, the neural representations of to-be-attended auditory streams are sharpened relative to unattended streams (Ding & Simon, 2012) in human non-primary auditory cortex (Ding & Simon, 2011; Mesgarani & Chang, 2012).

The neural representation of a sound’s fundamental frequency, called the frequency-following response (FFR; Kraus, Anderson, & White-Schwoch, 2017), conveys information related to pitch processing (Gockel, Carlyon, Mehta, & Plack, 2011) and is also measurable with EEG and MEG. Although the FFR is sometimes referred to as the “brainstem response,” it has multiple origins including the auditory brainstem, thalamus, and cortex (Coffey, Herholz, Chepesiuk, Baillet, & Zatorre, 2016; Hartmann & Weisz, 2019; Tichko & Skoe, 2017; see Coffey, Nicol, et al., 2019 for discussion). Even a single EEG channel placed at the vertex or frontal scalp captures information about the fidelity with which the auditory system as a whole preserves useful sound information, making single-channel FFR an attractively accessible and widely used technique (Coffey, Nicol, et al., 2019).

The FFR is sensitive to individual differences in HIN perception (Anderson, Parbery-Clark, White-Schwoch, & Kraus, 2013; Brown & Bacon, 2010; Coffey et al., 2017), suggesting that the quality of feed-forward pitch encoding is important. While FFRs are typically assessed using evoked responses obtained from repeated presentation of an acoustic stimulus (i.e., a tone or a speech syllable; Kraus et al., 2017; Krizman & Kraus, 2019; Skoe & Kraus, 2010), recent work shows that high-frequency acoustic information can also be reconstructed from electrophysiological responses to continuous input (Forte et al., 2017; Maddox & Lee, 2018). In a selective listening paradigm using two competing speakers, small enhancements of the attended stream relative to the unattended stream were reported (Etard, Kegler, Braiman, Forte, & Reichenbach, 2019; Forte et al., 2017). These results agree with findings that the gain of pitch representations in the FFR is accessible to top-down attentional mechanisms (Hartmann & Weisz, 2019). Few studies have looked at the relationship between neural representations in both frequency bands, which is relevant to understanding how and when streams of task-relevant acoustic information are separated from background noise and enhanced.

The paradigms used in HIN studies differ in many ways, including the nature of the target and ignored sound streams, physiological signal measured, recording equipment used, and the presence of additional cues in the stimuli. Although the majority of HIN experimental tasks and clinical tasks operationalize HIN perception in simple unimodal auditory terms (e.g., sentences in broadband noise, two-talkers reading different stories), it has been well documented that other cues that are frequently present in real-world HIN conditions contribute to performance. Spatial information improves stream segregation and HIN performance (Carhart, Tillman, & Johnson, 1968; Divenyi & Oliver, 1989; Yost, Sheft, & Dye, 1994), as does the presence of visual information that is congruent with the attended sound source (Crosse, Di Liberto, & Lalor, 2016; Golumbic, Cogan, Schroeder, & Poeppel, 2013; Puschmann et al., 2019). Visual information enhances auditory perception, and helps to resolve auditory perceptual ambiguity (Golumbic et al., 2013), likely by priming the brain to receive relevant input at specific times. HIN skills are also affected by knowledge of language syntax and semantics (Golestani, Rosen, & Scott, 2009; Pickering & Garrod, 2007), familiarity with the speaker’s vocal timbre (Barker & Newman, 2004; Souza, Gehani, Wright, & McCloy, 2013; Yonan & Sommers, 2000), and prior knowledge of the target (Agus, Thorpe, & Pressnitzer, 2010; Bey & McAdams, 2002), which can be used to predict, constrain, and evaluate the interpretation of incoming information (Bendixen, 2014).

Recognizing that HIN is a complex skill involving multiple interacting neural systems to degrees that depend on the nature of the task and on individuals’ strengths (Jasmin, Dick, Holt, & Tierney, 2019; Yates, Moore, Amitay, & Barry, 2019), some experimental approaches divide HIN perception into its component skills and compare their relative contributions. For example, Coffey, Arseneau-Bruneau, Zhang, and Zatorre (2019) compared the benefits to HIN performance of offering spatial, visual, and predictive information (in separate conditions) to otherwise matched auditory stimuli presented in noise. Each additive cue conferred a benefit to listeners over unimodal auditory HIN performance; however, these benefits were also related differently to individuals’ experiences with musical training, multilingualism, and both measures of top-down (i.e., auditory working memory) and bottom-up (i.e., fine pitch discrimination) skills. While reductionist approaches offer insight into individual differences and specific processes, a complementary experimental approach in which multiple cues are simultaneously present could more closely replicate the brain’s integrative approach to naturally occurring HIN situations, in which multiple cues are often present. A notable aspect of this design is that it uses simple musical melodies, which reduces the influence of inter individual differences in linguistic skills, and enables precise balancing of sensory conditions in a relatively naturalistic framework. Task performance nonetheless correlated with a sentence-based measure of HIN (i.e., the HIN task; Nilsson, Soli, & Sullivan, 1994), suggesting overlap in neural mechanisms, and supporting the use of musical stimuli in HIN studies.

### The Current Study

The main focus of this work is to better understand how acoustic information in the two frequency bands described above is enhanced through processes of selective attention, under difficult but cue-rich listening conditions. Previous work has shown greater stream differences in later (higher level) cortical brain areas, suggesting that only attended information is carried forward from early auditory regions (e.g., Du & Zatorre, 2017; Puschmann, Baillet, & Zatorre, 2018). We hypothesized that while both the envelope and the fundamental frequency reconstruction of the attended stream would be greater than that of the unattended stream (as shown in previous work), the slower responses that are thought to originate from primary, secondary, and later auditory cortical regions would show greater stream-specific enhancements than the FFR, which comes from subcortical regions, and at the level of the auditory cortex, likely only comes from early regions (Coffey et al., 2016; Hartmann & Weisz, 2019). We recorded single-channel EEG while listeners were asked to follow a target stream of music embedded within four other sound streams, including one with equivalent but temporally rearranged (i.e., scrambled) acoustic information and three with scrambled information at different timbres and spectral ranges. The target sound stream was thus concealed in a rich cacophony of sound, constituting both energetic and informational masking. Listeners were offered visual, spatial, and predictive cues simultaneously in addition to auditory information. We decoded the attended, ignored, and total sound streams within each frequency band from the EEG signals, analyzed their temporal properties, and compared their relative strengths.

While decoding accuracy is most effective using multiple EEG channels, it is not known whether the single-channel technique can be used to decode FFR information (Mirkovic, Debener, Jaeger, & De Vos, 2015). A secondary aim was to test whether single-channel decoding is possible in both frequency ranges, which, due to its experimental simplicity, opens many possibilities for measuring larger samples and more sensitive populations.

## MATERIALS AND METHODS

### Participants

Data were collected from 18 subjects who were participating in an undergraduate-level course on experimental methods for musical neurocognition (average age = 22.1 years, *SD* = 1.6, range = 21–26). Three subjects were left-handed, and 15 subjects were female. Subjects participated on a voluntary basis as part of their training, after providing written informed consent in accordance with protocols reviewed by the ethics committee of the University of Montreal. All subjects had pure tone hearing thresholds better than 20 dB SPL (sound pressure level) in both ears at octave frequencies between 125 Hz and 8 kHz (with one exception who had a marginally elevated threshold of 25 dB SPL in the right ear at 6,000 Hz only). All subjects reported having no neurological conditions nor other difficulties with hearing or vision.

Information regarding the subjects’ musical experience was collected via the Montreal Music History Questionnaire (Coffey, Herholz, Scala, & Zatorre, 2011) and is reported for completeness; because our convenience sample is of modest size and is highly musically and linguistically heterogeneous, we do not attempt statistical analyses of finer grained relationships between pitch representation and experience in sound herein. Sixteen subjects reported being native French speakers, one was a native Russian speaker, and one was a native Arabic speaker. Eight subjects were monolingual French speakers and 10 were bi- or trilingual. Only one spoke a tonal language, and two believed themselves to have absolute pitch. On average, subjects had 1,969 cumulative musical practice and training hours (*SD* = 3,621, range = 0–15,023) and started training at age 7.57 (*SD* = 3.25, range = 3–16).

### Stimulation

#### Attended, ignored, and background streams

As in previous work (Disbergen, Valente, Formisano, & Zatorre, 2018; Pressnitzer, Suied, & Shamma, 2011), we take advantage of music as a excellent platform to study complex stream segregation and the integration processes that appear to be common to both music and language. To ensure that the results would be relevant across both domains, we created musical stimuli with temporal properties that have been strongly related to language processing.

**Figure 1.**
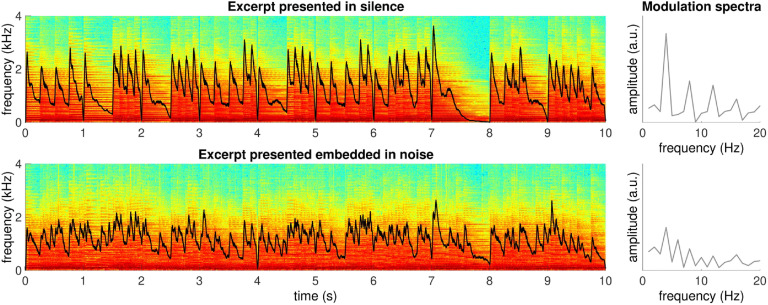
Spectrograms of 10 s excerpts of the stimulus presented in silence (top) and embedded in the background streams (bottom). Spectral amplitude summed over frequencies, which represents how sound intensity fluctuates over time, is superimposed as black curves, the modulation spectra of which are shown at right. The stimulus presented in silence has a clear modulation peak in the amplitude envelope at 4 Hz (top right), whereas it is obscured by the addition of background noise (bottom right).

The target stream consisted of a musical excerpt of an instrumental work by Georg Philipp Telemann (1681–1767), Sonata for Flute in F(TMV 41:F2, mvmt 1). Only the first 10 measures of the melodic line were used (21 s). The rate of tone onsets was 120 quarter notes or 240 eighth notes per min, corresponding to 2 and 4 Hz. The strongest frequency was 4 Hz, confirmed by analyzing the frequency content of the spectral amplitude envelope (Ding et al., 2017; see Figure 1). Cortical response in the 1–4 Hz range reliably predicts speech recognition in the presence of background noise (Ding, Chatterjee, & Simon, 2014). In recordings of naturally occurring speech and musical recordings, there are small differences in the average peak frequency of amplitude envelope modulations (i.e., 5 Hz for speech and 2 Hz for music; Ding et al., 2017). Our tone rate falls close to the peak of the speech range, to which the human auditory system is highly sensitive (Teng & Poeppel, 2019), maximizing the likelihood of engaging mechanisms that are also active in speech-in-noise processing.

The melodic excerpt was converted to midi electric piano timbre using Musescore (www.musescore.com) to provide strong attacks (sudden sound onsets), which produce robust responses from the auditory system. The excerpt was then transposed such that all tones had fundamental frequencies below ∼500 Hz, so as to be centred on the human vocal pitch range and maximize the auditory system’s response (Tichko & Skoe, 2017). The final range of fundamental frequencies was 104–277 Hz. We split measures in half, resulting in 20 new measures, each containing 2 beats and between 1 and 8 notes. Measures were exported as separate files (WAV, sampling frequency 44,100 Hz). Because the decay of the rendered piano tones exceeds the measure and would have resulted in mixed acoustic information between measures, measures were trimmed to 1 s and windowed using a 10 ms raised cosine ramp, using custom scripts (MATLAB; www.mathworks.com). The right channel was copied over the left channel to ensure identical frequency information was presented to each ear. The acoustic properties of each ear and for the attended (in which measures were presented sequentially, in their original order) and ignored sound stream (in which measures were presented in a scrambled order) were therefore acoustically comparable, except at longer timescales (>1 s).

This experimental design strongly challenges the auditory system with high levels of informational and energetic masking yet offers multiple additional cues. By using musical stimuli that have frequency information within the ranges of interest for speech, while removing the complications of multiple layers of linguistic processing, we are able to emphasize enhancements via top-down processing and multimodal integration on acoustic representation.

#### Attentional control

In each trial, one of the six quarter notes in the musical excerpt was randomly replaced by a triplet in which the first and last notes were the same pitch, and the central note was raised by a tone. These targets occurred in the attended stream for 50% of trials and in the unattended stream for the other 50% of trials; subjects were asked to indicate (on a score sheet) after each trial if they had heard a triplet in the attended stream, and an accuracy score of correct hits and correct misses was calculated as an attentional control (Disbergen et al., 2018). Triplets occurred only in the auditory modality; the visual representation was that of the original excerpt, such that the task could not be accomplished using only visual information. The first three subjects received an earlier version of the triplet manipulation in which each of the triplets had the same pitch—the subjects expressed frustration with the task (although they performed above chance levels). The central note was raised by a tone for the remainder of the subjects (*N* = 15) to make the variation more salient.

#### Spatial cues, visual cues, and predictive information

**Figure 2.**
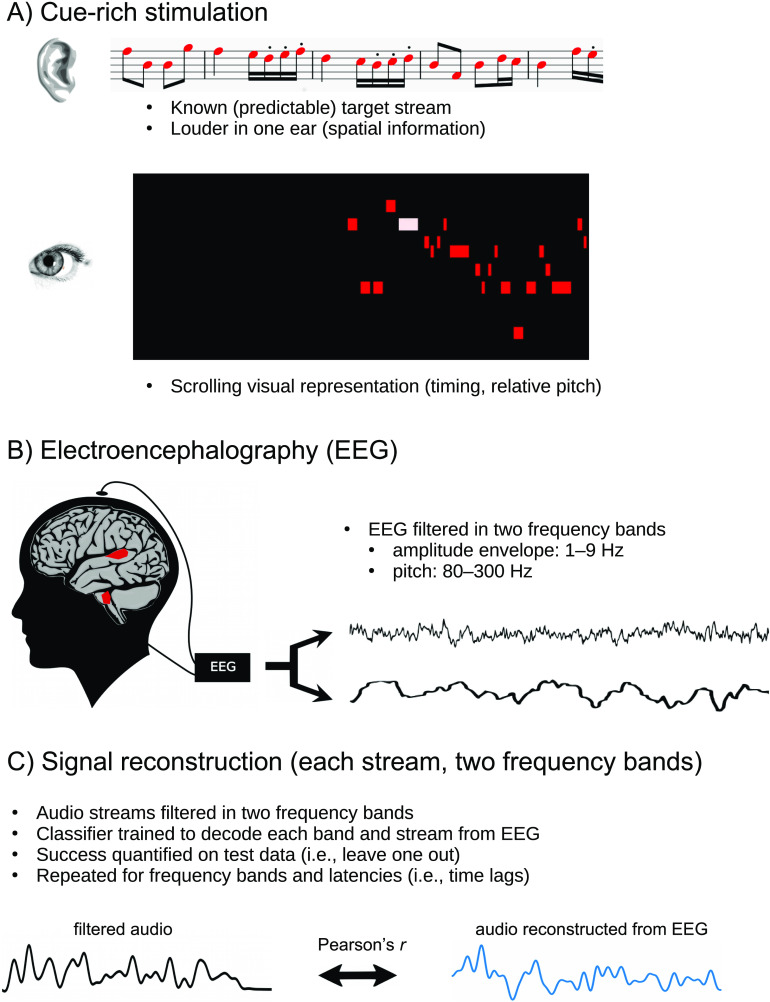
A) Auditory stimulation consisted of a mixture of a to-be-attended stream, an acoustically similar but scrambled to-be-ignored stream, and three streams of additional background noise. Predictive, spatial, and visual cues were provided simultaneously. B) Electroencephalography (EEG) was collected and filtered in two information-bearing frequency bands. C) Reconstruction of sound envelope and frequency-following response was performed using ridge regression to establish a linear backward mapping between EEG neural responses and the sound streams. The measure of successful reconstruction is an average correlation between the EEG-based reconstruction and the auditory information.

To simulate spatial separation between different sound sources, the attended and unattended streams were presented at a higher sound level in one ear than the other (L > R by *0.8 or R > L by *0.8, corresponding to a perceptual experience of the sound originating approximately 45 degrees to one side or the other of a straight-ahead position). A visualization was prepared using a Python clone of a freely available musical animation software (www.musanim.com; see Figure 2). Two versions of the video were then created (using Kdenlive; https://kdenlive.org/en/) in which the animation was reduced in size and moved either to the left or right side of the screen to provide a spatial cue as to whether the attended sound would be at a higher level in the left or right ear.

Prior to the EEG recording, subjects were familiarized with five demonstration versions of the stimuli and encouraged to practice listening for triplets both with no background noise and in noise. At the beginning of each block, subjects heard the original musical excerpt presented in silence three times binaurally, with the visual representation, to refresh their memory and facilitate use of top-down predictive cues.

#### Background noise

Three background streams were included to provide a consistent level of “multi-music” background noise, and to reduce the perception of salient or disturbing coincidences created by the offset between to-be-attended and to-be-ignored streams (e.g., if the unattended stream was jittered by half a beat on a given trial, it might have been perceived as a new “fused” melody at double the original tempo). The background streams also served to reduce temporal glimpsing (i.e., when a target stimulus is momentarily unmasked; see Vestergaard, Fyson, & Patterson, 2011), in an analogous fashion to multitalker speech used in some speech-in-noise tasks (e.g., Wilson, 2003).

The three background streams were created by transposing the original melody either up or down by a major or minor second and changing their timbre, which produced background tones with fundamental frequencies in the frequency range of 87–294 Hz. This manipulation ensured that the attended and unattended streams were well buried in the noise and could not be separated solely by paying attention to high or low pitch ranges; these background streams were played at a reduced volume (*0.6 amplitude with respect to the attended and ignored streams) and equally in both ears, such that it would theoretically be possible to separate the to-be-ignored stream from the rest of the background noise based on sound level, timbre, and spatial separation. The timbral manipulation was intended to simulate the observation from speech-in-noise research that familiarity with the speaker’s voice improves HIN perception (Barker & Newman, 2004; Souza et al., 2013; Yonan & Sommers, 2000). The attended stream began very slightly ahead of the other streams in order to facilitate stream following. Each additional stream started within the duration of the first beat (0.025–0.475 s in steps of 0.005 s, randomized). The attended, ignored, and background streams were combined using custom scripts to create 78 unique stimuli (see Table 1).

**Table 1.** Stimulus design

Streams	Order of measures	Timbre	Key: note range (Hz)	L-R level balance	Visual representation	Jitter	Oddball targets
Attended	In order	Electric piano	D♭: A♭2–D♭4 (104–277 Hz)	L > R by *0.8 or R > L by *0.8; randomized	Yes, position reflects ear of greater volume (spatial cue)	Starts first	Yes, 50% of trials
Ignored	Scrambled	Electric piano	D♭: A♭2–D♭4 (104–277 Hz)	Opposite to target	No	Jittered by 25–500 ms in steps of 5 ms, randomized	Yes, opposite 50%
Background 1	Scrambled	Acoustic bass	B♭: F2–B♭3 (87–233 Hz) (transposed down by minor second)	Equal, *0.6 with respect to 1 and 2	No	Jittered by 25–500 ms in steps of 5 ms, randomized	No
Background 2	Scrambled	Recorder	D: A2–D4 (110–294 Hz) (transposed up by minor second)	Equal, *0.6 with respect to 1 and 2	No	Jittered by 25–500 ms in steps of 5 ms, randomized	No
Background 3	Scrambled	Vibraphone	C♭: G♭2–C♭3 (93–247 Hz) (transposed down by minor second)	Equal, *0.6 with respect to 1 and 2	No	Jittered by 25–500 ms in steps of 5 ms, randomized	No

#### Design

The experiment was divided into 3 blocks (8.5 min each). In each block, subjects listened to the 3 in-silence trials and 78 task trials. During a 3–4 s break after each trial, subjects indicated on a form whether they had heard a triplet in the attended stream. Subject comfort, compliance, and motivation were informally assessed for each subject and between each run; all subjects remained motivated and alert for the duration of the experiment.

### EEG Data Collection and Preprocessing

EEG data were recorded in a magnetically shielded audiometric booth from monopolar active Ag/AcCl electrodes placed at Cz (10–20 International System), and both mastoids, using an averaged reference (BioSemi; www.biosemi.com). Two ground electrodes were placed above the right eyebrow. Because active electrodes reduce nuisance voltages caused by interference currents by performing impedance transformation on the electrode, we confirmed that direct-current offset was close to zero during electrode placement instead of measuring impedance. Electrode signals were amplified with a BioSemi ActiveTwo amplifier, recorded using open filters with a sampling frequency of 2,048 Hz, and stored for offline analysis using BioSemi ActiView software. The auditory signal was simultaneously recorded into the EEG data in order to facilitate precise alignment of auditory stimulation with neural responses.

Data were preprocessed in EEGLAB (Delorme & Makeig, 2004) and then with custom MATLAB scripts (Mathworks; www.mathworks.com). EEG data were band-pass filtered both from 1–9 Hz for sound envelope reconstruction and from 80–300 Hz for fundamental response (default order); both outputs were down-sampled to 1,000 Hz, and re-referenced to the average of the right and left mastoid channels. Trials were cut into 22 s epochs for reconstruction analysis. Amplitude envelopes of the musical streams were obtained using a Hilbert transform, followed by 1–9 Hz band-pass filtering. Filtering was performed using a third order Butterworth filter and the filtfilt function in MATLAB for zero-phase digital filtering of the data, as in Puschmann et al. (2018).

### Reconstruction Method

Reconstruction of the sound envelope and FFR was performed using the multivariate temporal response function toolbox for MATLAB (Crosse, Di Liberto, Bednar, & Lalor, 2016). Ridge regression was used to fit a linear backward mapping between EEG neural responses of the Cz channel and the sound stream. This model allows for stimulus reconstruction that incorporates a window of relative time lags between sound input and EEG response (e.g., 0–200 ms) or a reconstruction that is based on a single relative time lag (e.g., neural response at 10 ms). We employed both of these strategies to explore the magnitude and temporal evolution of reconstruction accuracy of different sound streams within the sound envelope and FFR bandwidths.

The regularization parameter, *λ*, was optimized for each sound stream and each subject with leave-one-out cross-validation across trials. This procedure uses grid values of *λ* (*λ* = 10^−2^, 10^−1^, …, 10^8^) and selects the optimal value based on mean squared error. Using the selected *λ* value, the model was trained on each of the trials to obtain regression weights. Model fitting for each sound stream was then performed with data from the selected trial and the mean regression weights obtained for this stream in all other trials. This leave-one-out parameter estimation procedure ensured that reconstruction did not depend on trial-specific properties of the recorded EEG data but was rather related to trial-independent mapping between the sound streams and neural response as measured by EEG (Puschmann et al., 2018). Pearson’s correlation, *r*, between the reconstructed and original sound stream was computed and averaged across trials to quantify the accuracy of the reconstruction. This process was repeated for each of the sound streams of interest (i.e., target, ignored, and entire sound stream), with audio and EEG data filtered within the 1–9 Hz and 80–300 Hz bandwidths. Given that the model may find spurious patterns in data, chance correlations between reconstructions and sound streams are likely to be above zero. We therefore computed chance *r* values by averaging values obtained by correlating the reconstruction of the target stream with the ignored sound stream, and the reconstruction of the ignored stream with the target sound stream. Thus, we computed chance values by attempting the reconstruction correlation with the wrong training data (O’Sullivan et al., 2014).

#### Calculation of reconstruction accuracy

The models for sound envelope (1–9 Hz) reconstruction included time lags between 0 and 200 ms, a range selected based on previous work to encompass the strongest response, thought to originate in Heschl’s gyrus and the planum temporale (see Puschmann et al., 2018, Figure 3B; see also Ding & Simon, 2012; Steinschneider, Liégeois-Chauvel, & Brugge, 2011). Models for FFR (80–300 Hz) included time lags between 0 and 35 ms. We performed a series of Wilcoxon signed-rank tests to compare the overall mean reconstruction of each of the target, ignored, and entire sound streams with chance values, as well as between attended and ignored streams, and each of attended and ignored streams with the entire sound stream. We report uncorrected *p* values, as well as *p* values corrected for false discovery rate (FDR) of multiple testing under dependency (Benjamini & Yekutieli, 2001).

#### Temporal evolution

We explored the temporal evolution of reconstruction accuracy (i.e., timing of the neural contributions to reconstruction) in each of the sound envelope and FFR frequency bands. For the former, we fit single lag models ranging from 0 to 200 ms for the attended, ignored, and entire sound stream, as well as for chance reconstruction values, as previously described. For the fundamental response, we fit single lag models ranging from 0 to 75 ms. Although the majority of the signal is expected to be <35 ms, reflecting the shorter expected time course of this signal (Etard et al., 2019), the cortical FFR response does not peak until ∼60 ms (Coffey et al., 2016), for which reason we explored a longer window. In these models each point in time represents how well sound can be reconstructed from the EEG neural response with contributions of the neural response from only a single lag. We smoothed reconstruction accuracy across lags in these models by using time lags of ±3 ms.

In each of the frequency domains and single time lags, the attended, ignored, and entire sound streams were compared to chance reconstruction using paired Wilcoxon signed-rank tests, and *p* values were corrected for the multiple comparisons across each of the time lags.

#### Enhancement of attended stream across frequency bands

To evaluate whether neural enhancement of the attended stream relative to the entire sound stream was greater in earlier, finer sound representations vs. in later ones, we used feature scaling (min-max normalization between 0 and 1) on attended stream and entire sound streams, for FFR and envelope values separately. We then calculated difference scores for each frequency band (i.e., attended minus entire streams, between −1 and +1), then statistically compared the FFR vs. envelope (Wilcoxon signed rank test, two-tailed).

## RESULTS

### Attentional Control

All subjects appeared alert and reported being engaged in the task, although subjects varied in their perceived difficulty with the attentional task, and three subjects had accuracy scores close to chance levels. As a precaution, we confirmed that conclusions of the study would not be affected by the exclusion of the three poorly performing subjects; results were qualitatively unaffected.

The mean accuracy of triplet identification across the entire sample was 63% (*SD* = 11; range = 47–82%); excluding the three subjects who received an earlier variant of the manipulation, the mean was 62%, (*SD* = 11); and the chance level was 50%). A one-sample Kolmogorov–Smirnov test was significant, indicating a nonnormal distribution of scores. A one-sample Wilcoxon signed-rank test showed that the group’s performance was above chance levels of accuracy, *Z* = 150, *p* = 5.02e-04 (one-sided, alpha = 0.05).

### Sound Envelope Reconstruction

#### Reconstruction accuracy

**Figure 3.**
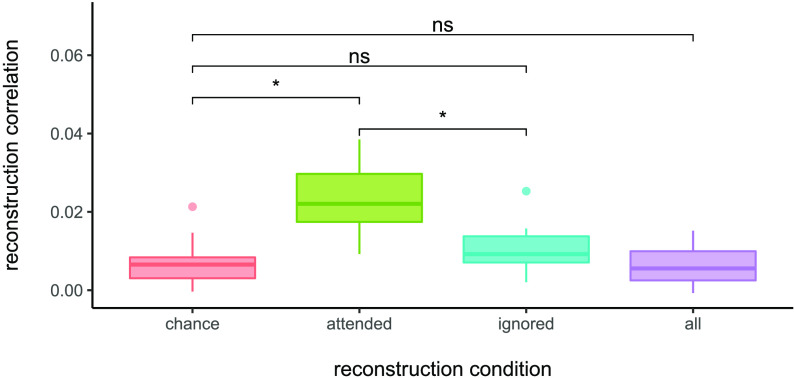
Sound envelope reconstruction accuracy. EEG data and auditory information were band-pass filtered between 1–9 Hz, and reconstruction included time lags of 0–200 ms. Reconstruction correlations are computed by taking average correlations between the EEG-based reconstruction and the auditory information across trials. Results from statistical analyses are shown for the attended, ignored, and entire sound streams, as well as for chance reconstruction correlations; for complete pairwise comparisons see Table 2.

The mean reconstruction accuracy of the attended stream within the window of interest (0–200 ms) was 0.023 (*SD* = 0.0086); the ignored stream was 0.0065 (*SD* = 0.0095); and the entire sound stream was 0.0039 (*SD* = 0.0065); see Figure 3. The mean reconstruction accuracy achieved by chance (i.e., the average of correlating the reconstruction of the attended stream with the ignored stream, and vice-versa) was 0.0034 (*SD* = 0.0075).

Of the attended stream, ignored stream, and entire sound stream, only the attended stream was reconstructed above chance levels (see Table 2). The attended stream was reconstructed significantly better than the ignored steam and the entire sound stream. To ensure that the results were robust to variations in the lag range selected, we also ran the analysis for the 0–500 ms range; this resulted in slightly higher reconstruction accuracies but did not change the pattern of results.

**Table 2.** Sound envelope reconstruction accuracy comparisons

Sound streams		Test statistic	*p* value	FDR-corrected *p* value
**Attended**	ignored	167	0.000053	0.00026*
**Attended**	chance	171	0.0000076	0.000056*
Ignored	chance	116	0.20	0.72
Entire sound stream	chance	102	0.50	1.00
**Attended**	entire sound stream	171	0.0000076	0.000056*
Ignored	entire sound stream	99	0.58	1.00

*Note*. In case of statistically significant results in pairwise analyses, the better reconstructed stream is indicated in bold font. FDR = false discovery rate.

* *p* < 0.05

#### Temporal evolution

**Figure 4.**
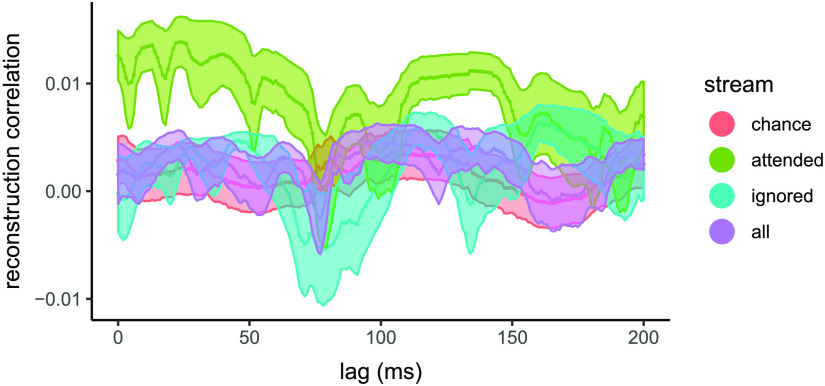
Temporal evolution of sound envelope reconstruction accuracy. Average reconstruction correlations across trials using single relative time lags between auditory information and neural response are shown for attended and ignored streams, entire sound stream, and for correlations obtained by chance. Models were fit for relative time lags of 0–200 ms. Shading indicates standard error.

We conducted a series of reconstructions at specific lags (0–200 ms), in order to explore the speech envelope reconstruction accuracy as a function of stream and relative time lag between sound input and EEG response. The attended stream showed two broad peaks, at approximately 40 ms and 130 ms (see Figure 4).

### Fundamental Frequency Reconstruction

#### Reconstruction accuracy

**Figure 5.**
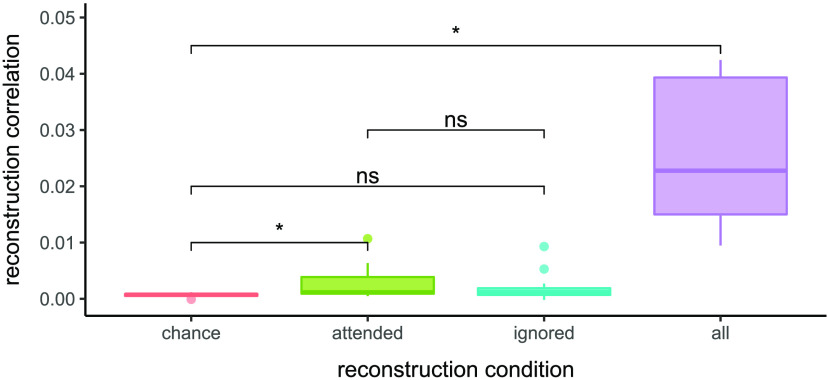
Frequency-following response reconstruction accuracy. EEG data and auditory information were band-pass filtered between 80–300 Hz, and reconstruction included time lags of 0–35 ms. Reconstruction correlations represent average correlations between the EEG-based reconstruction and the auditory information across trials. Results from statistical analyses are shown for the attended, ignored, and entire sound stream, as well as for chance reconstruction correlations; for complete pairwise comparisons see Table 3.

In the high frequency range (80–300 Hz), within a window of interest (i.e., neural response lags from 0–35 ms), the mean reconstruction accuracy of the attended stream was 0.0023 (*SD* = 0.0028); the ignored stream was 0.0015 (*SD* = 0.0025); and the entire sound stream was 0.022 (*SD* = 0.015; see Figure 5. The mean reconstruction accuracy achieved by chance (i.e., the average of correlating the reconstruction of the attended stream with the ignored stream, and vice versa) was −0.00055 (*SD* = 0.0013).

All three streams (i.e., attended, ignored, and the entire sound stream) were constructed above chance levels (see Table 3). The entire sound stream was reconstructed significantly better than any of the other streams. The attended stream was not constructed with greater accuracy than the ignored stream.

**Table 3.** Frequency-following response reconstruction accuracy comparisons

Sound streams		Test statistic	*p* value	FDR-corrected *p* value
Attended	ignored	119	0.15	0.38
**Attended**	chance	161	0.00033	0.0012*
**Ignored**	chance	149	0.004	0.012*
**Entire sound stream**	chance	168	0.000038	0.00019*
Attended	**entire sound stream**	3	0.000038	0.00019*
Ignored	**entire sound stream**	3	0.000038	0.00019*

*Note*. In case of statistically significant results in pairwise analyses, the better reconstructed stream is indicated in bold font. FDR = false discovery rate.

* *p* < 0.05

#### Temporal evolution

**Figure 6.**
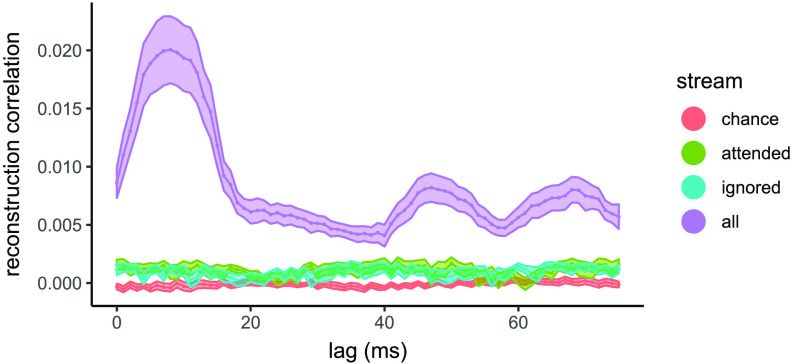
Temporal evolution of frequency-following (fundamental) response reconstruction accuracy. Average reconstruction correlations across trials using relative time lags ranging from 0 to 75 ms and including ±3 ms between auditory information and neural response are shown for attended and ignored streams, entire sound stream, and for correlations obtained by chance. Shading indicates standard error.

The reconstruction of the entire stream showed one main peak at 8 ms and two smaller peaks at approximately 50 ms and 70 ms (see Figure 6). The results were statistically significant for most of the window (FDR-corrected *p* value = 0.05).

#### Enhancement of attended stream across frequency bands

**Figure 7.**
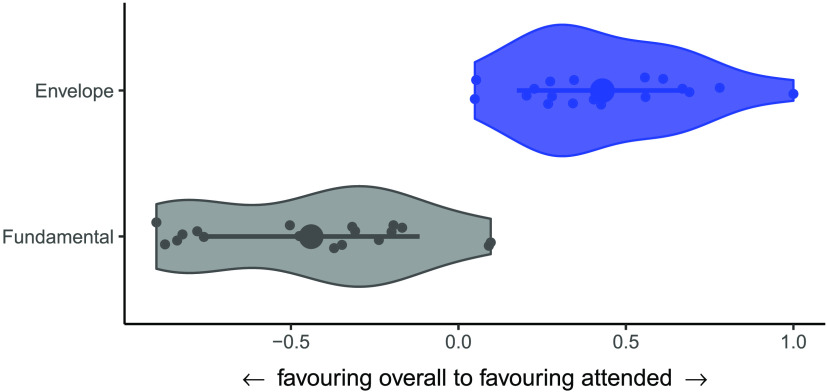
Enhancement of attended stream differs across frequency bands. In the frequency-following response, the overall stream is represented to a greater extent in neural activity than the to-be-attended stream, whereas in the envelope-following response, the attended stream is enhanced relative to the entire sound stream. Feature scaling was conducted to enable the comparison (i.e., scaling each set of values between 0 and 1).

Comparison of the FFR and envelope reconstruction accuracies revealed a significant difference in the relative representation of the attended and overall sound streams (Wilcoxon signed-rank test, two-tailed, *Z* = −3.72, *p* < 0.0002; see Figure 7).

## DISCUSSION

### Difficulty Level and Attentional Manipulation

The main objective of our study was to test whether sharpening of the neural representations of sound across frequency bands is a plausible mechanistic explanation under highly challenging but cue-rich HIN. The overall above-chance results from the attentional control task (i.e., triplet detection) suggest both that participants were engaged in the task and able to successfully attend to the target stream and that an appropriately high level of difficulty was achieved; this confirmation served as a basis for further exploring differences in neural correlates’ strength of representation of the various sound streams.

### Reconstruction Accuracy of the Sound Envelope

Previous studies on speech perception with competing sound streams reported greater reconstruction accuracy of target vs. ignored streams’ envelopes, suggesting that the brain is able to enhance task-relevant (to-be-attended) auditory information (e.g., Mesgarani & Chang, 2012; Puschmann et al., 2018). In agreement with previous results, we found that when using musical stimuli, the to-be-attended stream was enhanced relative to the unattended streams. Interestingly, the entire sound stream’s envelope was not represented above chance levels, and neither was the to-be-ignored stream. In contrast to previous designs that mostly used two competing sound streams, our design included a to-be-ignored stream that was acoustically identical except in the ordering of musical measures, which were scrambled to limit predictive information, and a great deal of background noise, which filled the gaps and limited the depth of envelope modulation of the entire sound stream (see Figure 1).

Taking a finer grained look at the time courses of the reconstruction, we find that the majority of information about the attended stream comes from two broad peaks in the neural representation, centred at approximately 40 and 120 ms (no clear above-chance peaks are observed in the unattended stream and in the entire sound stream). These results are generally consistent with the time courses observed previously using speech stimuli (i.e., stories; Puschmann et al., 2018) and provide further evidence that the same neural mechanisms are likely employed in this music-based task as in speech-based tasks (see also Coffey, Arseneau-Bruneau, et al., 2019). However, as compared with previous results, the pattern of reconstruction accuracies over lags is less clear. Differences in paradigm design and data acquisition may account for this observation. As compared with previous work, our design includes a more extreme degree of sound degradation/masking.

A limitation of the present design is that we do not have a visual-only condition or a no-visual-cue condition and can therefore not assess whether part of the reconstruction performance of the attended stream might be accounted for by the visual cues alone. We speculate that the nature of the visual cue, in which the viewer sees representations of ∼20 notes scrolling across the screen at a time (Figure 2A), would not drive the visual system strongly in a rhythmic manner, but rather the coupling of the visual and auditory systems might confer a benefit. More work is needed to clarify at which auditory processing level the visual system’s influence is apparent in continuous listening paradigms.

Previous work showed that one advantage of expertise comes from musicians’ ability to better represent the ignored sound stream, perhaps thereby being better able to suppress it (Puschmann et al., 2018). An explanation for the result that the unattended stream was not decoded is that our convenience sample is not comprised of subjects with sufficiently high levels of expertise with sound to accurately represent the competing streams. The nature of the stimuli and the task itself likely also play a role. As described above, our competing stimuli applied both a high degree of energetic and informational masking, yet offered little in the way of longer-range predictive cues (i.e., on the order of seconds), which are present in simultaneously presented stories (e.g., vocal timbre and pitch, semantic and syntactic cues). The busy background sound stream also lacked clear amplitude fluctuations that might offer temporal glimpses of the to-be-ignored stream by itself that might be used to successfully follow the to-be-ignored stream (Vestergaard et al., 2011).

### Reconstruction Accuracy of the Fundamental Frequency

In the present study, we were able to reconstruct the fundamental frequencies (80–300 Hz) of the attended and ignored streams as well as for the entire sound stream above chance levels. The mean reconstruction accuracy of the attended stream was numerically higher than the ignored stream, but in contrast to Etard et al. (2019), we did not observe a statistically significant attentional enhancement. In Etard et al., the mean ratio of attended to unattended correlations was 1.2, in contrast to 1.5 in the present study, suggesting that a difference in statistical sensitivity due to the recording methods (64 channel EEG vs. single channel) or statistical analysis (different multiple comparison correction) could account for the discrepancy. The reconstruction accuracy for the fundamental frequency of the entire sound stream was much more readily decoded than single streams. An explanation for this observation could be that the entire stream simply contained more pitch-related information than the monophonic streams, as pitch information was continuously present. Conversely, pitch information was only present when tones were played (and not in between notes) in the monophonic streams, resulting in proportionally less encoded pitch information.

An alternative explanation for both observations concerns the degree to which stream segregation has occurred in the observed signals. Noting that fundamental frequency reconstruction works well for the entire scene in this study, it is possible that the attended and unattended streams are not well segregated at this early processing level because the attended stream is continually embedded in rich background noise. Selective attention to a single stream relies on successful object formation, which can fail for a variety of reasons, such as high levels of energetic masking, sources with similar spectrotemporal structure, and ambiguous or conflicting cues (Shinn-Cunningham, 2008)—all of which are present in the present paradigm. Attention effects may appear at earlier processing levels in the relatively easier two-talker design (Etard et al., 2019), when the acoustic information in the attended stream can be frequently glimpsed through gaps in the ignored stream and the streams can also be grouped by other acoustic differences in the speakers’ voices. This explanation would parallel conclusions from Puvvada and Simon (2017), which showed that in the envelope frequency band, earlier processing stages (<80 ms) represent the entire acoustic scene, whereas later responses represent separate elements of the scene, at which stage ignored information may be actively repressed (Khalighinejad, Herrero, Mehta, & Mesgarani, 2019). It is therefore possible that the difficulty level (e.g., of the two-talker speech paradigm used previously and the present 5-stream music-in-noise design) affects the degree and processing level at which attention effects manifest, due to differences in the effectiveness of bottom-up object formation (Shinn-Cunningham, 2008).

The temporal evolution of the entire sound stream was clearly reconstructed across latencies, with the strongest peak at about 8–9 ms. This latency is consistent with much of the FFR literature, which shows a maximum cross-correlation lag between stimulus and response at about 9 ms (Forte et al., 2017; Krizman & Kraus, 2019), and roughly agrees with recent speech reconstruction results, which found one peak at 9 to 12 ms (Etard et al., 2019). Unlike in that study, we also found two later peaks after 40 ms, the latency of which imply cortical FFR responses (Coffey et al., 2016; Tichko & Skoe, 2017). Taken together, these results suggest that the reconstruction methods are capturing the same brain responses to continuous stimuli that are normally recorded in an evoked response paradigm, which is most often used to study FFR and has been related to a broad set of individual differences and behaviours (Kraus et al., 2017; Skoe & Kraus, 2010).

### Comparison of Attentional Enhancement Across Frequency Bands

Direct comparison of the relative strength of attended vs. entire sound stream representation across frequency bands showed that whereas the attended stream only comprises a small fraction of the encoded fundamental frequency information, it dominates the sound envelope representation. This pattern of results suggests a progressive shift in representation within the auditory system from encoding sound information gathered from the outside world with relatively high fidelity, to selectively sharpening and enhancing relevant information at lower frequencies that occurs with greater time lags and likely reflects downstream processes. The notion of progressive refinement of information would not be a foreign concept in the auditory system. For example, Parras et al. (2017) found that neurons along the auditory pathway exhibit a hierarchical organization of prediction error whereby prediction errors are detectable in subcortical regions but increase in strength as the information moves towards the auditory cortex.

While there is considerable evidence of predictability of sound enhancing or sharpening neural representations, an alternative idea is that prediction errors are the key factor in successful hearing-in-noise perception. Using multivariate fMRI decoding, Blank and Davis (2016) showed that expectations in the form of prior knowledge suppressed rather than enhanced auditory representations, under HIN conditions. Specific to the FFR (but not under HIN conditions), Gorina-Careta, Zarnowiec, Costa-Faidella, and Escera (2016) showed that responses to a repeated speech syllable (/wa/) evoked decreased neural responses when they were highly predictable.

However, using continuous speech stimuli, Etard et al. (2019) did find an enhancement of the fundamental frequency, and while our results are not significant, they point in the same direction. These results raise the question of whether the nature and degree of predictability found in naturalistic, continuous stimuli such as spoken narratives or musical stimuli has the same effect on neural representation as does, for example, repeating a syllabic stimulus hundreds of times. The conditions under which fundamental frequency representations are enhanced or suppressed as a means of achieving HIN perception remains for further work, but our results argue in favour of considering the nature of the stimuli and the stimulation paradigm carefully.

### Multiple Cues

The current study allowed us to examine the effect of attention on auditory representations under difficult but cue-rich conditions. Its design does not allow us to attribute enhancements separately to the spatial, visual, and predictive cues offered, nor to explore differences between the effectiveness with which people can take advantage of different cues. Behavioural tasks such as the Music-In-Noise Task (MINT) can be used to further study how integrating information across neural systems results in improved performance, the benefits of which can differ according to one’s experience with sound—MINT results differed according to musical and linguistic experience (Coffey, Arseneau-Bruneau, et al., 2019) and correlated with results from well-used language-based task (Nilsson et al., 1994). Our results also support the use of instrumental musical stimuli, which are devoid of linguistic cues and can be easily manipulated to alter their informational content while holding acoustic features constant.

### Extending the Capabilities of Single-Channel EEG

Single-channel EEG has been an important part of FFR work for decades (mostly using token repetition paradigms), due to its simplicity, effectiveness, and low imposition on the subject (Coffey, Nicol, et al., 2019; Skoe & Kraus, 2010). Previous work on speech envelope decoding has shown that accuracy is best with 25 EEG channels (Mirkovic et al., 2015), and is most effective around frontotemporal electrodes (O’Sullivan et al., 2014; Puschmann et al., 2017), but whether single-channel EEG could be used in decoding neural responses to continuous-listening paradigms was an open question (Mirkovic et al., 2015). The present results clearly demonstrate decoding of auditory information above chance levels in both envelope and pitch frequency ranges from a single channel, from about 20 min of data per participant. While future work will likely improve decoding accuracy from single-channel montages by optimizing electrode placement and data analysis, we propose that single-channel EEG in the current configuration can already be useful to study the auditory system in naturalistic listening conditions.

### Conclusions

The present work is novel in that it looks at neural representation of multiple sound streams within and across frequency bands, in a challenging but cue-rich listening situation in which attended and ignored streams are acoustically matched, and it uses a musical cocktail party paradigm rather than the conventional speech-in-noise paradigm, to our knowledge for the first time. The results contribute evidence that one of the ways in which HIN perception is accomplished is via active, selective, and progressive enhancement of the to-be-attended stream. While finer, earlier representations of a stimulus’ fundamental frequency show little effect of attentional enhancement, later, slower, neural representations are dominated by the attended stream. In comparison to other work and in agreement with aggregate results showing heterogeneity of experience-related enhancements on HIN tasks, our results suggest that it is the *nature* of the HIN task that affects which neural mechanisms are employed and contribute the most to neural representations and overall perceptual performance. Our results also demonstrate that single-channel EEG is sufficient to reconstruct both low- and high-frequency band information, which we hope will facilitate larger studies and studies in populations that do not easily support more involved methods (i.e., high density EEG, MEG). Future work on HIN mechanisms could further explore the influence of training, expertise, and individual differences on neural representations, and should consider the type and extent of masking sound, as well as the availability of multimodal and predictive cues, as mechanisms of enhancement may apply contextually. We suggest that music-in-noise paradigms such as this may be a strong complement to speech-based studies, as they can be tightly acoustically/visually controlled, yet appear to rely on the same neural mechanisms, and can allow us to remove the influence of individual differences in language proficiency (van Wijngaarden, Steeneken, & Houtgast, 2002). An emerging perspective on evoked responses, particularly the FFR, considers the auditory system (including cortical and subcortical components) as an integrated whole that operates over multiple frequencies (e.g., envelope and fundamental frequency bands) and timescales (e.g., tens of ms to hundreds of ms; Kraus et al., 2017). This idea can be extended to other brain systems that interact with it for a more complete understanding of human perception, as visual, memory, and attentional systems influence the strength of envelope and fundamental frequency neural representations.

## ACKNOWLEDGMENTS

The authors would like to acknowledge the International Laboratory for BRAin, Music and Sound Research (BRAMS) for equipment access; Mihaela Felezeu, Alexandre Lehmann, and Marc Schönweiser for technical assistance on audio and EEG equipment, and Isabelle Peretz for supporting our ethics application; Lars Hausfeld for advice on the reconstruction analysis; Giovanni Beltrame for assistance with video creation and consultation on feature scaling; Robert Zatorre for comments on an early draft; and Simon Rigoulot and his course students for their enthusiastic participation. SP was supported by a research fellowship of the German Research Foundation (Deutsche Forschungsgemeinschaft). EC was supported by a CGS Vanier Scholarship. KG was supported by an NSERC USRA scholarship.

## FUNDING INFORMATION

Sebastian Puschmann, Deutsche Forschungsgemeinschaft, Award ID: PU590/1-1.

## AUTHOR CONTRIBUTIONS

Keelin Greenlaw: Formal analysis; Methodology – lead; Visualization – lead; Writing – original draft; Writing – review & editing. Sebastian Puschmann: Formal analysis; Methodology; Supervision; Writing – review & editing. Emily B. J. Coffey: Conceptualization – lead; Data curation – lead; Formal analysis; Investigation – lead; Methodology; Project administration; Supervision – lead; Visualization; Writing – original draft – lead; Writing – review & editing – lead.
